# Aspartate aminotransferase-to-platelet ratio index as a novel predictor of early mortality in heat stroke patients: a multi-centre retrospective study

**DOI:** 10.1080/07853890.2025.2478485

**Published:** 2025-03-15

**Authors:** Min Wang, Yun Li, Yuan Cao, Meng-Meng Yang, Fu-Jing Liu, Jie Jiao, Sheng-Yuan Wang, Bin Song, Lu Wang, Yi-Qi Wu, Hong-Jun Kang

**Affiliations:** ^a^ Medical School of Chinese PLA, Beijing, China; ^b^ Department of Critical Care Medicine, the First Medical Centre, Chinese PLA General Hospital, Beijing, China; ^c^ Department of Emergency, The Affiliated Changzhou NO.2 People’s Hospital of Nanjing Medical University, Jiangsu, China; ^d^ Department of Critical Care Medicine, Hainan Hospital of Chinese PLA General Hospital, Sanya, China; ^e^ The Sixth Medical Centre, Chinese PLA General Hospital, Beijing, China; ^f^ The Seventh Medical Centre, Chinese PLA General Hospital, Beijing, China; ^g^ Department of Nephrology, First Medical Center of Chinese PLA General Hospital, Nephrology Institute of the Chinese People’s Liberation Army, National Key Laboratory of Kidney Diseases, National Clinical Research Center for Kidney Diseases, Beijing Key Laboratory of Kidney Disease Research, Beijing, China

**Keywords:** Heat stroke, APRI, predictor, mortality

## Abstract

**Background:**

The aspartate aminotransferase-to-platelet ratio index (APRI) is an effective non-invasive marker for chronic liver dysfunction. Given that heat stroke patients often suffer from poor prognosis due to multi-organ involvement, with liver injury and coagulation dysfunction being of particular concern, this study aims to investigate whether APRI can comprehensively reflect liver injury and coagulation dysfunction in heat stroke patients and explore its relationship with 28-day mortality.

**Methods:**

This retrospective study analysed electronic medical records from patients treated at 57 grade A tertiary hospitals in China from May 2005 to May 2024. The primary outcome was 28-day mortality, and the secondary outcome was 7-day mortality. Restricted cubic splines (RCS) were utilized to visualize the relationship between APRI and 28-day mortality risk. The independent association between APRI and outcomes was assessed using Cox proportional hazards models, with multivariable analyses controlling for confounding factors. The predictive ability of APRI for outcomes was evaluated using receiver operating characteristic (ROC) curves.

**Results:**

A total of 450 eligible patients were included, with 71 deaths occurring within 28 days. RCS analysis showed a positive correlation between APRI and 28-day mortality. Participants were divided into higher (APRI ≥ 15.14) and lower (APRI < 15.14) APRI groups. Cox proportional hazards models indicated that individuals with higher APRI had a significantly increased 28-day mortality rate (HR 5.322, 95% confidence interval [CI] 2.642-10.720, *p* < 0.0001). Subgroup and interaction analyses confirmed the robustness of the core findings. Additionally, the areas under the ROC (AUROC) for APRI predicting 28-day mortality was 0.823 (95% CI 0.772–0.875), significantly higher than the AST to ALT ratio (0.526, 95% CI 0.448–0.605) and total bilirubin (0.694, 95% CI 0.623–0.765).

**Conclusion:**

APRI is an independent predictor of early mortality risk in heat stroke.

## Introduction

Heat stroke is a severe heat-related illness often accompanied by multi-organ dysfunction, including the brain, liver, kidneys and lungs [1]. Among the organs affected by heat stroke-induced multi-organ dysfunction, the liver is considered one of the first to sustain damage [2]. Acute liver injury and its more severe form, acute liver failure, are complications of heat stroke and direct causes of mortality in heat stroke patients [3–6]. Studies have shown that liver failure may occur more frequently than expected, with most patients succumbing within about a week of onset [7,8]. Elevated liver enzymes are a significant predictive factor and closely related to the occurrence of multi-organ failure [9]. Multiple studies have revealed that elevated liver enzymes are an independent risk factor for heat stroke prognosis [2,4,9]. Liver enzymes aspartic aminotransferase (AST) and alanine aminotransferase (ALT) typically rise rapidly from 24 h to the third day after onset, beginning to decrease around the fifth day. However, this decline does not indicate alleviation of liver injury but may result from the depletion of liver enzymes following massive hepatocyte death [2]. This underscores the importance of prediction of later disease progression through early elevated liver enzymes. Although changes in liver enzymes can reflect the degree of liver dysfunction following heat injury, no formally identified biomarkers currently exist to quantify the extent of liver injury or recovery post-heat stroke [10]. Therefore, exploring an easily accessible biomarker that can comprehensively and sensitively reflect liver injury is urgently needed.

Platelets (PLT) are implicated in the multi-organ dysfunction process of heat stroke by regulating inflammation, maintaining tissue integrity and defending against infections, with particular concern regarding coagulation dysfunction [11–13]. Studies have found that thrombocytopenia is closely associated with poor outcomes in sepsis [14]. Furthermore, mean platelet volume (MPV) and PLT ratio have been identified as promising early predictors of mortality in critically ill septic patients [15]. As an independent risk factor for poor prognosis in heat injury and even heat stroke, low PLT is associated with liver damage in heat-related illnesses [16,17]. Approximately 71% of heat stroke patients experience thrombocytopenia [18]. When core temperatures exceed 40 °C, relative reductions in platelet count greater than 30% below baseline can occur, and absolute reductions are observed at temperatures exceeding 41 °C [19]. The severity of coagulation dysfunction in heat stroke patients is strongly correlated with poor prognosis [20].

Aspartate Aminotransferase-to-Platelet Ratio Index (APRI) is a diagnostic marker for liver fibrosis and cirrhosis in patients with non-alcoholic fatty liver disease or hepatitis C, demonstrating superior performance as a non-invasive indicator of liver function [21]. It has been widely recognized and recommended by the World Health Organization [22,23]. Liver fibrosis and cirrhosis are closely associated with early inflammatory stimuli [24]. As heat stroke represents an acute inflammatory response syndrome, APRI may exhibit more rapid and intense changes compared to chronic inflammatory processes such as cirrhosis. A high APRI value may indicate significant liver damage and potential coagulation dysfunction in heat stroke patients, thereby reflecting poor prognosis and higher mortality rates. This study aims to explore the potential of APRI in predicting mortality in heat stroke patients.

## Materials and methods

### Data source

This retrospective study analysed clinical data from patients diagnosed with heat stroke across 57 hospitals in China, spanning from May 2005 to May 2024. The diagnosis of heat stroke was based on the Expert Consensus on the Diagnosis and Treatment of Heat Stroke in China [25]. Inclusion criteria required a history of exposure to high-temperature or high-humidity environments, or intense physical exertion, combined with one or more of the following clinical manifestations: central nervous system dysfunction (such as coma, seizures, delirium, or abnormal behaviour), a core body temperature exceeding 40 °C, multiple organ dysfunction (≥2) (liver, kidney, striated muscle, gastrointestinal tract, etc.), and severe coagulopathy or clinical signs of Disseminated Intravascular Coagulation (DIC).

Patients were excluded if they were under 18 years of age, had pre-existing liver or kidney insufficiency, hematologic disorders, or if their hospital stay was less than 24 h. Patients with missing values for either AST or PLT were also excluded. This study followed the guidelines of the Transparent Reporting of a multivariable prediction model for Individual Prognosis Or Diagnosis (TRIPOD) Statement [26].

### Ethical considerations

This study was strictly retrospective and conducted anonymously. The Research Ethics Committee of the Chinese PLA General Hospital approved this study (approval number and date, NOS2023-803 and March 28, 2024), and the need to obtain informed consent was waived because all data were de-identified. This study was conducted in accordance with the ethical standards of the Committee for Responsibility in Human Experiments and the 1975 Declaration of Helsinki.

### Data collection

**Figure 1.**
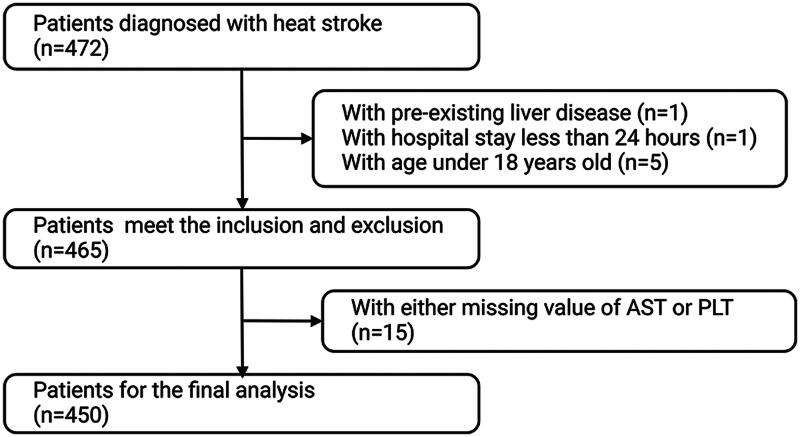
Flow chart of study participant selection procedure.

We retrospectively collected data from the electronic medical records platform of 473 patients diagnosed with heat stroke. Based on the inclusion and exclusion criteria, 450 patients were ultimately included in this study (Figure 1). Effective cooling was defined as a body temperature below 38.5 °C upon hospital admission, accompanied by cooling interventions implemented between symptom onset and hospital arrival, including ice pack application, cold water immersion, and fan cooling. We collected demographic information, including gender and age; vital signs, including body temperature (T), heart rate (HR), respiratory rate (RR) and mean arterial pressure (MAP); and laboratory indicators, including routine blood tests (hemoglobin (Hb), white blood cell count (WBC), neutrophil ratio (Neur) and PLT), organ function biochemical indicators (AST, ALT, serum albumin (ALB), total bilirubin (TBIL), direct bilirubin (DBIL), serum creatinine (Cr), lactate dehydrogenase (LDH), creatine phosphokinase (CK), creatine kinase isoenzyme (CK-MB), and blood urea nitrogen (BUN)), brain natriuretic peptide (BNP), coagulation function (prothrombin time (PT), activated partial thromboplastin time (APTT), thrombin time (TT), fibrinogen (Fib) and D-dimer (D-D)). Additionally, we recorded whether invasive mechanical ventilation (IMV) or vasoactive drugs (adrenaline, noradrenaline, dopamine, dobutamine) were used, and whether the patient experienced coma. All vital signs and laboratory test indicators represent the worst values recorded within 24 h of hospital admission, and therapeutic interventions and medications were also documented during this 24-hour period post-admission. We calculated APRI and the AST to ALT ratio (SLR). The primary outcome was 28-day survival post-admission, and the secondary outcome was 7-day mortality.

### Statistical analysis

Continuous variables conforming to a normal distribution are presented as mean ± standard deviation (SD), and group comparisons were made using the independent samples t-tests. For continuous variables not adhering to a normal distribution, median and interquartile ranges (IQR) were reported, with group comparisons performed *via* the Kruskal–Wallis test. Categorical variables are expressed as counts and percentages (%), with differences assessed using the chi-square test.

Using the ‘maxstat’ package and setting the minimum proportion for each group at 0.3, the optimal cutoff value for the continuous variable APRI was determined, dividing the study subjects into lower and higher APRI groups. Survival analysis was conducted using the Kaplan–Meier (KM) method to estimate the survival probabilities of heat stroke individuals based on different APRI levels, and the log-rank test was used to compare the two curves. Restricted cubic splines (RCS) were employed to explore the potential nonlinear relationship between APRI and 28-day mortality in heat stroke patients, testing 3, 4 and 5 knots. Ultimately, 4 knots were selected based on the smallest Akaike Information Criterion (AIC). RCS represent a smoothing technique for modelling nonlinear relationships between continuous predictors and response variables. This approach partitions the continuous variable into intervals defined by knots, fitting cubic polynomials within interior segments while enforcing linear constraints on the terminal segments. By reducing model complexity and mitigating overfitting, RCS preserve parsimony while ensuring that extrapolations beyond the observed data range follow biologically plausible linear trends [27,28]. In contrast, the ‘maxstat’ package in R identifies optimal cutoff points for continuous variables by leveraging maximally selected rank statistics, which iteratively evaluate potential thresholds to maximize between-group divergence. Specifically, for survival outcomes, the algorithm computes log-rank statistics across all feasible cutoffs, selecting the value that yields the most significant stratification. Conservative p-value adjustments, such as the Lausen-Schumacher method or conditional Monte Carlo simulations, are employed to address multiplicity and enhance inferential robustness [29].

The Cox proportional hazards model was used to assess the independent association between APRI and 28-day mortality in HS patients. Prior to modelling, we evaluated multicollinearity among confounding factors using variance inflation factor (VIF) analysis and correlation coefficient tests (Supplementary Figure 1). Results were presented for Model 1, Model 2 (adjusted for age and gender), and Model 3 (adjusted for age, gender, WBC, ALT, ALB, TBIL, Cr, LDH, CK-MB, BUN, BNP, PT, APTT, TT, Fib and the presence of coma).

To determine the applicability of APRI across subgroups and identify differences in APRI within different patient populations, we conducted subgroup analyses based on age, gender, T, Hb, WBC, ALT, ALB, TBIL, Cr, LDH, CK-MB, BUN, PT, TT, Fib and SLR. Stratified and interaction analyses were conducted considering variables such as gender (female or male), age (<65 and ≥65 years), T (<40 and ≥40 °C), Hb (≥110 and <110 g/L), WBC (<10 and ≥10 × 10^9/L), ALT (≤40 and >40 U/L), ALB (≥40 and <40 g/L), TBIL (≤23 and >23 mmol/L), Cr (≤133 and >133 μmol/L), LDH (≤450 and >450 U/L), CK-MB (≤24 and >24 U/L), BUN (≤7.1 and >7.1 mmol/L), PT (≤15 and >15 s), TT (≤18 and >18 s), Fib (≥2 and <2 g/L) and SLR (≤1.5 and >1.5). The receiver operating characteristic (ROC) curve was used to evaluate the accuracy of APRI in predicting survival outcomes in heat stroke patients. Statistical analyses were performed using Python (version 3.9.16) and R (version 4.3.2). A two-sided *P*-value of less than 0.05 was considered statistically significant.

## Results

### Baseline characteristics of study participants

A total of 450 participants met the criteria for the current study (Figure 1). The optimal APRI cutoff value for survival was 15.14, dividing the participants into two groups: the higher APRI group (APRI ≥ 15.14, *N* = 159) and the lower APRI group (APRI < 15.14, *N* = 291) (Supplementary Figure 2). Compared to the lower APRI group, individuals in the higher APRI group exhibited several significant differences. Firstly, the 28-day mortality rate was higher in the higher APRI group (35%) compared to the lower APRI group (5%). The higher APRI group had a lower proportion of effective early cooling, higher body temperatures, and higher rates of invasive mechanical ventilation and vasoactive drug use (*p* < 0.001). Additionally, they showed elevated levels of ALT, AST, WBC, TBIL and DBIL (Table 1).

**Table 1.** Baseline characteristics between lower APRI group and higher APRI group.

Variables	ALL[*N* = 450]	Lower APRI[*N* = 291]	Higher APRI[*N* = 159]	P
Male, n (%)	380 (84.4)	235 (80.8)	145 (91.2)	0.005
Age(years), M [Q1, Q3]	28.0 [21.0;50.0]	31.0 [21.0;54.0]	24.0 [20.5;42.0]	0.001
BMI, mean (SD)	23.7 (2.2)	23.7 (2.1)	23.8 (2.4)	0.790
Effective cooling, n (%)	224 (49.8)	165 (56.7)	59 (37.1)	<0.001
T(°C), M [Q1, Q3]	40.0 [39.1;41.0]	40.0 [39.0;41.0]	40.6 [39.6;41.5]	<0.001
HR(beats/min), M [Q1, Q3]	99.0 [80.0;123]	94.0 [78.0;120]	109 [86.0;140]	<0.001
RR(beats/min), M [Q1, Q3]	20.0 [18.0;27.0]	20.0 [18.0;26.0]	22.0 [18.0;30.0]	0.041
MAP(mmHg), M [Q1, Q3]	87.0 [74.0;96.2]	88.0 [77.0;96.8]	85.0 [69.0;94.5]	0.014
Hb(g/L), M [Q1, Q3]	127 [113;138]	130 [118;139]	120 [104;136]	<0.001
WBC(*10^9/L), M [Q1, Q3]	12.4 [8.63;16.1]	11.6 [7.90;15.4]	13.2 [10.3;18.4]	<0.001
Neut, M [Q1, Q3]	87.0 [79.6;91.1]	85.4 [75.8;89.9]	90.3 [85.6;93.7]	<0.001
PLT(*10^9/L), M [Q1, Q3]	99.5 [40.4;170]	143 [98.0;190]	31.0 [21.0;50.5]	<0.001
ALT(U/L), M [Q1, Q3]	106 [35.0;489]	48.0 [25.3;110]	902 [322;2546]	<0.001
AST(U/L), M [Q1, Q3]	166 [60.1;679]	78.0 [40.2;158]	1189 [540;2717]	<0.001
ALB(g/L), mean (SD)	36.0 (5.77)	37.1 (5.60)	33.9 (5.51)	<0.001
TBIL(mmol/L), M [Q1, Q3]	26.8 [17.6;45.0]	21.3 [15.4;31.9]	48.3 [29.3;97.9]	<0.001
DBIL(mmol/L), M [Q1, Q3]	12.5 [8.13;22.3]	10.6 [6.40;14.9]	24.5 [12.9;46.8]	<0.001
Cr(mmol/L), M [Q1, Q3]	126 [94.0;177]	113 [84.9;143]	171 [126;232]	<0.001
LDH(U/L), M [Q1, Q3]	458 [290;1137]	329 [248;468]	1410 [820;2429]	<0.001
CK(U/L), M [Q1, Q3]	1423 [482;5723]	716 [301;2789]	4330 [1718;12454]	<0.001
CK-MB(U/L), M [Q1, Q3]	28.5 [10.5;68.0]	20.0 [9.30;44.0]	54.6 [20.5;149]	<0.001
Myo(ng/ml), M [Q1, Q3]	804.6 [275.4,2561.8]	392.6 [148.5,1024.1]	1886.0 [732.3,4214.2]	<0.001
TnT(ng/ml), M [Q1, Q3]	0.1 [0.0,0.3]	0.1 [0.0,0.2]	0.2 [0.1,0.5]	<0.001
TnI(pg/ml), M [Q1, Q3]	0.2 [0.1,0.9]	0.2 [0.0,0.5]	0.6 [0.2,2.4]	<0.001
BUN(mmol/L), M [Q1, Q3]	7.34 [5.51;9.54]	6.91 [5.08;8.88]	8.10 [6.90;10.5]	<0.001
BNP(pg/ml), mean (SD)	2558 (18451)	1529 (4211)	4442 (30485)	0.233
PT(s), M [Q1, Q3]	16.5 [13.9;24.5]	14.8 [13.0;16.8]	28.8 [20.3;50.3]	<0.001
APTT(s), M [Q1, Q3]	40.0 [30.2;62.8]	34.3 [28.0;42.5]	65.0 [48.1;119]	<0.001
TT(s), M [Q1, Q3]	20.2 [17.1;33.1]	18.7 [16.6;21.6]	32.2 [19.4;60.8]	<0.001
Fib(g/L), M [Q1, Q3]	1.99 [1.57;2.53]	2.17 [1.81;2.79]	1.52 [0.92;2.10]	<0.001
D-D(ng/ml), M [Q1, Q3]	2.44 [0.80;6.63]	1.33 [0.62;4.19]	5.26 [2.82;18.7]	<0.001
IMV, n (%)	177 (39.3)	79 (27.1)	98 (61.6)	<0.001
Coma, n (%)	253 (56.2)	169 (58.1)	84 (52.8)	0.331
Vasoactive drugs, n (%)	106 (23.6)	53 (18.2)	53 (33.3)	<0.001
APRI, M [Q1, Q3]	4.39 [0.96;38.4]	1.45 [0.59;3.92]	70.6 [33.8;216]	<0.001
SLR, M [Q1, Q3]	1.40 [0.97;2.11]	1.46 [1.05;2.19]	1.28 [0.86;2.02]	0.041
7-days death, n (%)	42 (9.3)	8 (2.7)	34 (21.7)	<0.001
28-days death, n (%)	71 (15.8)	15 (5.15)	56 (35.2)	<0.001

BMI, body mass index. T, temperature. HR, heart rate. RR, respiratory rate. MAP, mean arterial pressure. Hb, hemoglobin. WBC, white blood cell count. Neur, neutrophil ratio. PLT, platelet. ALT, alanine aminotransferase. AST, aspartic aminotransferase. ALB, serum albumin. TBIL, total bilirubin. DBIL, direct bilirubin. Cr, serum creatinine. LDH, lactate dehydrogenase. CK, creatine phosphokinase. CK-MB, creatine kinase isoenzyme. Myo, myoglobin. TnT, troponin T. TnI, troponin T. BUN, blood urea nitrogen. BNP, brain natriuretic peptide. PT, prothrombin time. APTT, activated partial thromboplastin time. TT, thrombin time. Fib, fibrinogen. D-D, D-dimer. IMV, invasive mechanical ventilation. SLR, AST to ALT ratio.

### Association between APRI and 28-day mortality in patients with heat stroke

The 28-day mortality rate was 15.78% (*N* = 71). RCS analysis revealed a positive nonlinear association between APRI and 28-day mortality in heat stroke patients (*p* for nonlinear = 0.0006) (Supplementary Figure 3). In the unadjusted analysis (Model 1), each unit increase in APRI was associated with a 0.6% higher risk of mortality (HR 1.006, 95% CI 1.005–1.007, *p* < 0.001) (Table 2). After adjusting for demographic factors (Model 2), the association remained robust (HR 1.006, 95% CI 1.005–1.008, *p* < 0.001). In the fully adjusted model (Model 3), which accounted for various clinical parameters, the association between continuous APRI and mortality remained stable (HR 1.006, 95% CI 1.003–1.009, *p* < 0.001) (Table 2).

**Table 2.** HRs (95% CIs) For 28-days mortality across groups of APRI.

Characteristic	Model 1		Model 2		Model 3	
	HR(95%CI)	*P* value	HR(95%CI)	*P* value	HR(95%CI)	*P* value
28 days mortality						
APRI*	1.006(1.005,1.007)	<0.0001	1.006(1.005,1.008)	<0.0001	1.006(1.003-1.009)	<0.0001
APRI group						
Low APRI	ref		ref		ref	
High APRI	8.131(4.597,14.380)	<0.0001	8.879(4.975,15.848)	<0.0001	5.322(2.642,10.720)	<0.0001

model 1: unadjusted. model 2: adjusted gender, age. model 3: adjusted gender, age, WBC, ALT, ALB, TBIL, Cr, LDH, CK-MB, BUN, BNP, PT, APTT, TT, Fib, coma.

*Continuous variable APRI.

**Figure 2.**
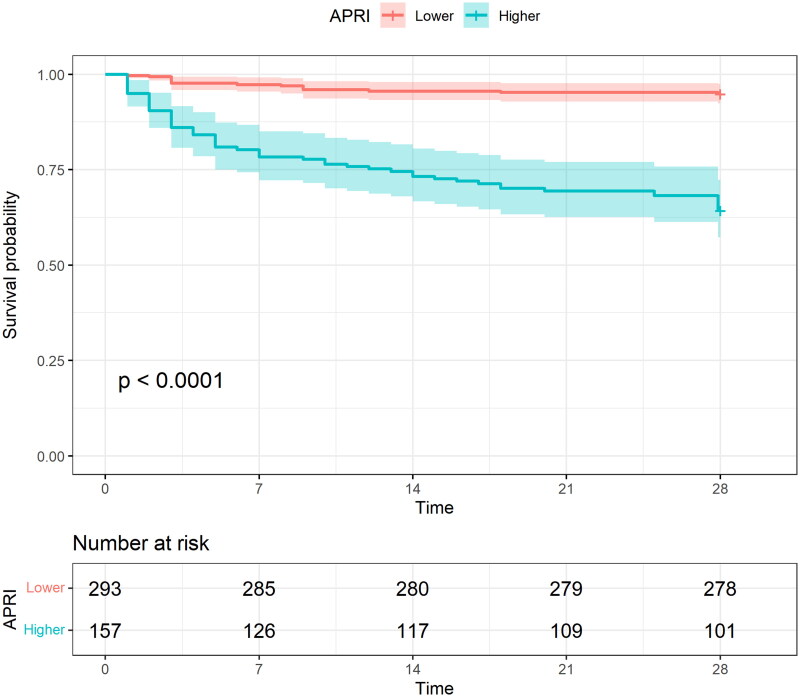
Kaplan–Meier Curve of 28-days survival rate with higher (≥15.14) and lower (<15.14) APRI values of patients with heat stroke.

**Figure 3.**
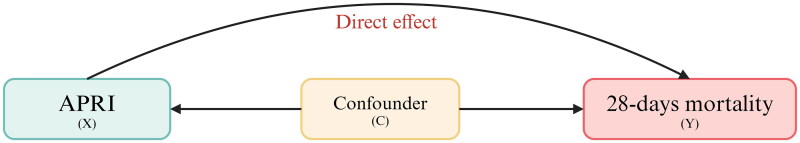
Directed acyclic graph (DAG) of APRI and 28-day mortality. Confounder: age, gender, WBC, ALT, ALB, TBIL, Cr, LDH, CK-MB, BUN, BNP, PT, APTT, TT, Fib.

Kaplan–Meier curve analysis showed that the survival rate was significantly lower in the higher APRI group compared to the lower APRI group (*p* < 0.0001) (Figure 2). When categorized using the optimal cutoff value, patients with high APRI had an 8.131-fold higher risk of mortality in the unadjusted analysis (Model 1: HR 8.131, 95% CI 4.597–14.380, *p* < 0.001). After adjusting for demographic factors, the association became slightly stronger (Model 2: HR 8.879, 95% CI 4.975–15.848, *p* < 0.001). However, in the fully adjusted model, the effect size was attenuated but remained significant (Model 3: HR 5.322, 95% CI 2.642–10.720, *p* < 0.001) (Table 2). Figure 3 shows the association of APRI with 28-day mortality and the confounding factors involved. Furthermore, we analysed the association between APRI and 7-day mortality (secondary outcome). The Kaplan–Meier survival curves (Supplementary Figure 4) and Cox regression analyses, both before and after adjusting for confounding factors (Supplementary Table 1), showed consistent trends with those observed for 28-day mortality.

Stratified analyses were conducted to evaluate the association between APRI and 28-day mortality across different subgroups (Supplementary Table 2). The elevated risk of mortality associated with high APRI remained consistent across most subgroups, though the magnitude of association varied. Notably, all subgroup analyses showed no significant interaction with APRI (*P* for interaction >0.05), indicating the prognostic value of APRI was generally consistent across different patient characteristics. However, marginally significant interactions were observed with ALB (*P* for interaction = 0.097) and PT (*P* for interaction = 0.056). Considering the limited sample size, these findings suggest that the association between APRI and mortality might vary by these parameters.

**Figure 4.**
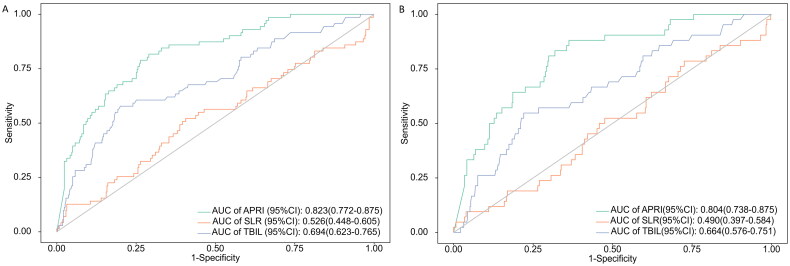
ROC Curves and AUC values of the APRI, SLR and TBIL for predicting 28 days (A) and 7 days (B) mortality for patients with heat stroke.

ROC analysis showed that AUROC for APRI in predicting 28-day mortality in heat stroke patients is 0.823, while the AUROC for SLR was 0.526 and for TBIL is 0.694. Additionally, for 7-day mortality in heat stroke patients, the AUROC for APRI was 0.804, for SLR was 0.490, and for TBIL is 0.664 (Figure 4). This indicates that APRI has significantly better accuracy in predicting mortality in heat stroke patients compared to SLR and TBIL, demonstrating good predictive performance for both 7-day and 28-day mortality in heat stroke patients.

## Discussion

This study comprehensively analysed the correlation between APRI and early mortality (28 days and 7 days) in heat stroke patients using various methods. By analysing clinical data from 450 heat stroke patients diagnosed across 57 hospitals in China, we identified a positive correlation between APRI and early mortality. Additionally, compared to commonly used indicators of liver dysfunction such as SLR and TBIL, APRI demonstrated effective predictive ability for both early mortality (28 days) and even earlier mortality (7 days). Furthermore, our study highlighted the significance of an APRI cutoff value greater than 15.14 for poor prognosis in heat stroke patients. To our knowledge, this is the first report identifying APRI as a predictor of early mortality in heat stroke.

The stable hazard ratio (HR = 1.006) for continuous APRI underscores its relatively independent association with mortality risk over 28-day periods. For categorical APRI, the HRs exhibited a notable increase following adjustment for age and gender (28-day mortality HR: from 8.131 to 8.879, 7-day mortality HR: from 8.785 to 9.688), but subsequently declined after further adjustment for additional clinical parameters (28-day mortality HR: from 8.879 to 5.322, 7-day mortality HR: from 9.688 to 4.525). This trajectory suggests that demographic factors, such as age and gender, as well as other clinical parameters, may act as confounding variables in the relationship between APRI and mortality risk in heat stroke patients. Younger patients’ physiological resilience and gender differences in heat injury responses may partially modulate this association [30].

The liver is considered one of the first organs to be affected by heat stress, yet liver dysfunction is not detected as early as dysfunction in other organs [2,31]. The pathophysiology of liver injury in heat stroke patients is complex. Common indicators of liver injury, such as TBIL, AST and ALT, reflect liver function status, but the liver’s compensatory mechanisms may maintain liver function early on, meaning these markers are not always directly related to liver injury [32]. Excessive inflammation and coagulopathy can further exacerbate liver function deterioration [33,34]. Conversely, hepatocyte damage can increase the risk of infection and coagulopathy due to reduced detoxification and protein synthesis, creating a vicious cycle that can be fatal for the patient [35]. Platelets also play a role in the inflammatory and thrombotic processes involved in this pathophysiology [36]. By incorporating PLT with AST, we aim to provide a more comprehensive reflection of the multiple pathophysiological processes involved in liver injury during heat stroke.

APRI was first proposed in 2003 and was formally recommended by the WHO as a non-invasive diagnostic tool for cirrhosis at the Asian Pacific Association for the Study of the Liver 2015 (APASL 2015) conference [23,37]. APRI has been widely promoted and applied in the diagnosis of chronic liver inflammation, such as liver fibrosis and cirrhosis. In recent years, it has also been studied in other fields, such as predicting cardiovascular risk, its relationship with colorectal tumor prognosis, and even predicting the severity of complex infections like dengue fever and haemorrhagic fever with renal syndrome [30,38–40]. Another study revealed that APRI is a potential early predictor of sepsis-associated liver injury in children [41]. These findings suggest that APRI can indicate multi-organ damage in various diseases. Furthermore, Fibrosis-4 (FIB-4), another widely used marker of liver fibrosis, has shown significant associations with adverse clinical outcomes in various acute critical conditions, including sepsis [28], acute kidney injury [42], metabolic disorders, and cancer[42], as well as with duration of mechanical ventilation and overall prognosis in COVID-19 patients[43,44]. These findings highlight that biomarkers traditionally associated with chronic liver dysfunction may hold valuable prognostic relevance in acute critical illnesses. Recent evidence further supports this perspective, demonstrating a significant association between APRI and in-hospital mortality in patients with sepsis-induced coagulopathy [45].

Given the overlapping pathophysiological features between heat stroke and sepsis – such as systemic inflammation, coagulopathy and multi-organ dysfunction – our study extends these observations by confirming APRI’s strong predictive value for early mortality in heat stroke patients. Heat stroke, a life-threatening condition characterized by widespread organ dysfunction, involves not only hepatic and coagulation abnormalities but also systemic inflammatory responses and dysfunction of other organs, particularly myocardial and skeletal muscle [46]. These systemic effects can influence APRI levels. Although our analysis adjusted for related variables (e.g. WBC, ALT, CK-MB, and PT) as potential confounders, the AST and PLT components of APRI remain intrinsically affected by dysfunction in other organs. Therefore, unlike its original application in chronic cirrhosis [47,48], changes in APRI in heat stroke cannot be exclusively attributed to liver dysfunction. The mechanisms underlying heat stroke-induced liver injury are intricately tied to systemic inflammatory responses, coagulation disturbances [34], abnormal hepatocyte apoptosis [49] and Kupffer cell dysfunction [50]. However, due to the multifaceted nature of heat stroke pathophysiology, many underlying mechanisms remain to be fully elucidated, underscoring the need for further research in this area.

Moreover, differences in the levels and extent of liver enzyme and inflammatory marker changes between acute and chronic liver dysfunction must be considered. In our study, the optimal cutoff value for APRI was defined as 15.14, which is significantly different from the maximum threshold of 2 used for cirrhosis. This observation highlights the unique characteristics of acute liver dysfunction. Severe systemic inflammatory response may be the primary driver in accelerating the progression of acute liver injury [51]. Typically, during an acute exacerbation, inflammation marker levels rise sharply, which intensifies as acute liver dysfunction worsens. The appearance and increasing levels of these inflammatory markers are closely linked to the heightened risk of liver dysfunction [52].

Our study demonstrates the robust predictive ability of APRI for early mortality risk in heat stroke patients. The advantage of using APRI as a prognostic predictor is that nearly all admitted patients, not just those with heat stroke, routinely undergo blood count and liver function tests, eliminating the need for additional efforts to obtain this indicator. While our current research shows that this correlation is most closely related to liver function and coagulation levels, the role of other pathophysiological processes requires further investigation and research.

However, our study has several limitations. First, despite utilizing electronic medical records from multiple hospitals, there may be variability in test results between different institutions. Second, we excluded patients who died within 24 h of admission, which might have influenced our findings since heat stroke is characterized by high early mortality. This exclusion, while necessary for complete laboratory data collection, means our results may not fully reflect APRI’s prognostic value in the most severe cases with rapid deterioration. Third, while we adjusted for potential confounding factors that might influence the relationship between APRI and mortality, we acknowledge that the range of adjusted confounders may not be exhaustive. Variables such as BMI, Myo, TnT, and TnI, which could further elucidate the association, were not included due to data limitations. Fourthly, due to the limitation of retrospective collection of case information, the cases in this study were not subdivided into exertional type and classic heat stroke. APRI may be different in different types of heat stroke, and further research is needed. Finally, as a retrospective study, our findings need to be validated by well-designed prospective studies to evaluate the predictive ability of APRI for mortality in heat stroke patients.

## Conclusion

This study reveals that APRI is a promising marker for predicting early mortality risk in heat stroke patients. This simple and readily available indicator can serve as a reference in clinical practice.

## Supplementary Material

Supplemental Material

## Data Availability

The dataset for this study can be made available for non-commercial research purposes upon reasonable request; please submit requests to the corresponding author. Each request will be evaluated to ensure compliance with ethical and legal restrictions governing the dataset.
