# Supplementary material for: Aspartate aminotransferase-to-platelet ratio index as a novel predictor of early mortality in heat stroke patients: a multi-centre retrospective study
Source: Ann Med. 2025 Mar 15;57(1):2478485. doi: 10.1080/07853890.2025.2478485 (PMC11912296; doi:10.1080/07853890.2025.2478485)

**Supplementary Materials**

**Aspartate Aminotransferase-to-Platelet Ratio Index as a Novel Predictor of Early Mortality in Heat Stroke Patients: A Multi-Center Retrospective Study**

Supplementary table 1. HRs (95% CIs) for 7-days mortality across groups of APRI.

| Characteristic | Model 1 | |  | Model 2 | |  | Model 3 | |
| --- | --- | --- | --- | --- | --- | --- | --- | --- |
|  | HR(95%CI) | P value |  | HR(95%CI) | P value |  | HR(95%CI) | P value |
| 7 days mortality |  |  |  |  |  |  |  |  |
| APRI* | 1.006(1.004,1.007) | <0.001 |  | 1.006(1.004,1.008) | <0.001 |  | 1.005(1.001-1.008) | <0.001 |
| APRI group |  |  |  |  |  |  |  |  |
| Low APRI | ref |  |  | ref |  |  | ref |  |
| High APRI | 8.785(4.066,18.980) | <0.001 |  | 9.688(4.428,21.197) | <0.001 |  | 4.525(1.739,11.769) | <0.001 |
| model 1: unadjusted. | | | | | | | | |
| model 2: adjusted gender, age. | | | | | | | | |
| model 3: adjusted gender, age, WBC, ALT, ALB, TBIL, Cr, LDH, CK-MB, BUN, BNP, PT,APTT, TT, Fib, coma. | | | | | | | | |
| *: Continuous variable APRI | | | | | | | | |

Supplementary table 2. Subgroup analysis of the associations between NLR and mortality among heat stroke.

| Characteristics | 28-days mortality | | | *P*  interaction |  | Characteristics | 28-days mortality | | | *P*  interaction |
| --- | --- | --- | --- | --- | --- | --- | --- | --- | --- | --- |
|  | Lower APRI | Higher APRI |  |  |  |  | Lower APRI | Higher APRI |  |  |
|  |  | HR(95%CI) | *P* |  |  |  |  | *HR(95%CI)* | *P* |  |
| Gender |  |  |  | NA |  | Cr |  |  |  | 0.540 |
| Male | Ref | 15.605(7.102,34.286) | <0.001 |  |  | <=133 | Ref | 8.007(3.151,20.346) | <0.001 |  |
| Female | Ref | NA | NA |  |  | >133 | Ref | 5.429(2.584,11.598) | <0.001 |  |
| Age |  |  |  | 0.798 |  | LDH |  |  |  | 0.454 |
| <65 | Ref | 8.523(4.645,15.641) | <0.001 |  |  | <=450 | Ref | 3.637(0.797,16.601) | 0.096 |  |
| >=65 | Ref | 5.933(1.000,35.600) | 0.051 |  |  | >450 | Ref | 7.188(2.874,17.976) | <0.001 |  |
| T |  |  |  | 0.830 |  | CK-MB |  |  |  | 0.863 |
| <40 | Ref | 6.037(1.509,24.142) | 0.011 |  |  | <=24 | Ref | 6.789(2.630,17.524) | <0.001 |  |
| >=40 | Ref | 7.290(3.878,13.700) | <0.001 |  |  | >24 | Ref | 7.615(3.587,16.163) | <0.001 |  |
| Hb |  |  |  | 0.240 |  | BUN |  |  |  | 0.224 |
| >=110 | Ref | 5.824(3.027,11.207) | <0.001 |  |  | <=7.1 | Ref | 12.268(4.454,33.791) | <0.001 |  |
| <110 | Ref | 14.409(3.421,60.686) | <0.001 |  |  | >7.1 | Ref | 5.672(2.840,11.328) | <0.001 |  |
| WBC |  |  |  | 0.752 |  | PT |  |  |  | 0.056 |
| <10 | Ref | 9.166(3.516,23.894) | <0.001 |  |  | <=15 | Ref | 5.075(1.653,15.586) | 0.005 |  |
| >=10 | Ref | 7.895(3.842,16.227) | <0.001 |  |  | >15 | Ref | 29.843(7.267,122.561) | <0.001 |  |
| ALT |  |  |  | NA |  | TT |  |  |  | 0.826 |
| <=40 | Ref | NA | NA |  |  | <=18 | Ref | 5.911(1.587,22.019) | 0.008 |  |
| >40 | Ref | 6.841(3.489,13.412) | <0.001 |  |  | >18 | Ref | 7.098(3.697,13.626) | <0.001 |  |
| ALB |  |  |  | 0.097 |  | Fib |  |  |  | 0.104 |
| >=40 | Ref | 26.916(5.946,121.852) | <0.001 |  |  | >=2 | Ref | 4.093(1.703,9.836) | 0.002 |  |
| <40 | Ref | 6.149(3.316,11.403) | <0.001 |  |  | <2 | Ref | 11.784(4.679,29.677) | <0.001 |  |
| TBIL |  |  |  | 0.148 |  | SLR |  |  |  | 0.222 |
| <=23 | Ref | 14.361(5.380,38.335) | <0.001 |  |  | <=1.5 | Ref | 13.666(4.812,38.810) | <0.001 |  |
| >23 | Ref | 5.940(2.898,12.172) | <0.001 |  |  | >1.5 | Ref | 6.485(3.201,13.138) | <0.001 |  |

**Supplementary figure legends**

Figure S1. Variance Inflation Factors (A) and Correlation Matrix (B) of Study Variables.


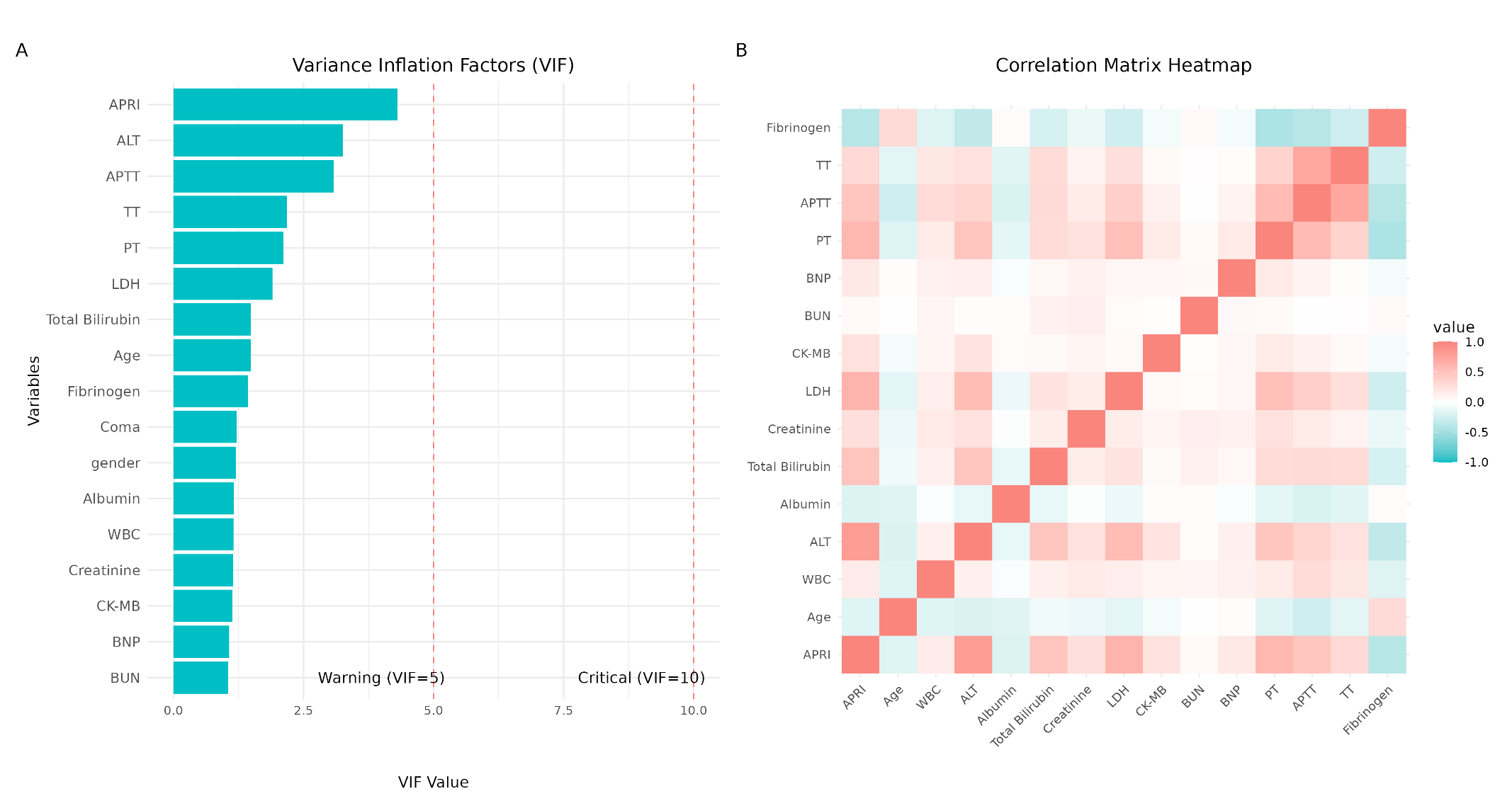


Figure S2. The cutoff point was calculated using the maximally selected rank statistics based on the ‘maxstat’ package.


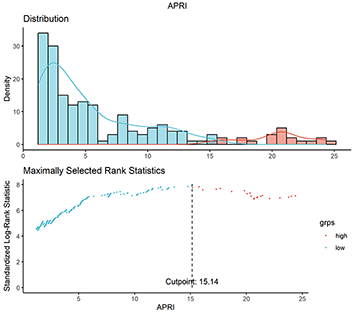


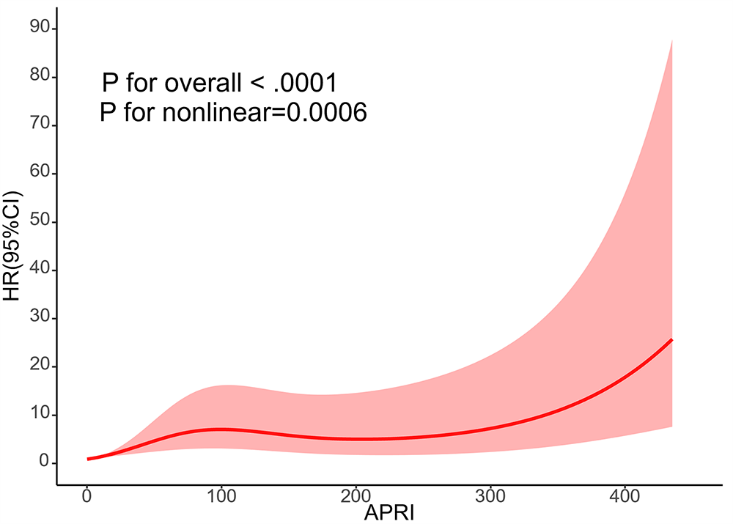
Figure S3. The association of APRI with 28-days mortality among heat stroke patients visualized by restricted cubic spline. Hazard ratios were adjusted for gender, age, WBC, ALT, ALB, TBIL, Cr, LDH, CK-MB, BUN, BNP, PT,APTT, TT, Fib and coma.

Figure S4. Kaplan-Meier Curve of 7-days survival rate with higher (>=15.14) and lower (<15.14) APRI values of patients with heat stroke.


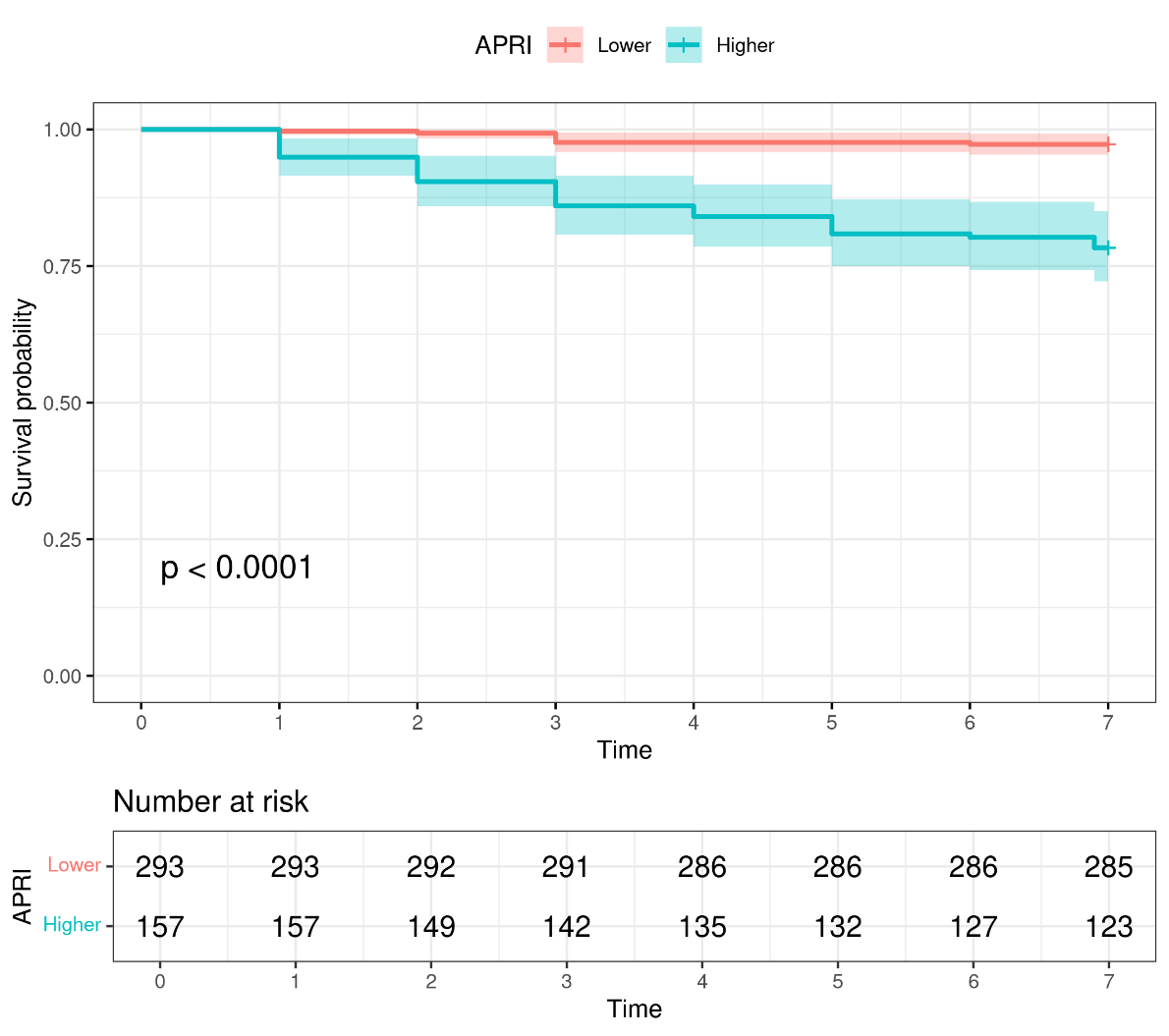

Supplement: Supplemental Material [file IANN_A_2478485_SM4033.docx]
